# Joining smallholder farmers’ traditional knowledge with metric traits to select better varieties of Ethiopian wheat

**DOI:** 10.1038/s41598-017-07628-4

**Published:** 2017-08-22

**Authors:** Chiara Mancini, Yosef G. Kidane, Dejene K. Mengistu, Melfa and Workaye Farmer Community, Amit, Letegabriel, Tsedal, B. Tinasu, Letekiros, Hadash, Letemedhin, A. Tinasu, Kiros, Leteberhan, Letekidan, Hareya, Letecheal, Hiritu, Ametezigu, Yohannes G. Amlak, Priest Gebre G. Slassie, Priest G. Selamma Girmay, G. Micheal Gebre, G. Slassie Mesfin, Kahsay Desta, Solomon Teklay, Haftu G. Kidan, Tesfay G. Egziabher, Priest Weldeslassie Desalegn, Hailemariam Gebre, Hiluf G. Micheal, Girmay Mebrahtu, Hailemichael, Amare Teklay, Esit Tesfay, Asrebeb Gitehun, Endale Tadesse, Mariye Asfaw, Kassaye Aragaw, Tegaye Brku, Yeshi Tadasse, Mariye Hailu, Adisse Kassun, Guzguz Gel aw, Melkam Emagn, Fenta Mitku, Asres Mengste, Bzunesh Yigzaw, Eset Tesfaw, Tesfaw Belay, Wodaje Yirga, Priest Agaju Sisay, Bewuketu Hailu, Priest Tefera Wale, Mulugeta Setegn, Tilaye Tesfie, Biset Meretie, Libay Kassie, Tegaye Biset, Yemataw Hailu, Libay Agazu, Mulatie Yigzaw, Adimasu Yigzaw, Getachew Abate, Mario Enrico Pè, Carlo Fadda, Matteo Dell’Acqua

**Affiliations:** 5 Melfa village (Kabele), Hagreselam district, Tigray, Ethiopia; 6 Workaye village (Kabele), Meket district, Geregera Amhara, Ethiopia; 1 Institute of Life Sciences, Scuola Superiore Sant’Anna, Pisa, Italy; 2 Sirinka Agricultural Research Center, Sirinka, Woldia Ethiopia; 3 Bioversity International, C/O International Livestock Research Institute (ILRI), Addis Ababa, Ethiopia; 4 Mekelle University, Department of Dryland Crop and Horticultural Sciences, Mekelle University, Mekelle, Ethiopia

## Abstract

Smallholder farming communities face highly variable climatic conditions that threaten locally adapted, low-input agriculture. The benefits of modern crop breeding may fail to reach their fields when broadly adapted genetic materials do not address local requirements. To date, participatory methods only scratched the surface of the exploitability of farmers’ traditional knowledge in breeding. In this study, 30 smallholder farmers in each of two locations in Ethiopia provided quantitative evaluations of earliness, spike morphology, tillering capacity and overall quality on 400 wheat genotypes, mostly traditional varieties, yielding altogether 192,000 data points. Metric measurements of ten agronomic traits were simultaneously collected, allowing to systematically break down farmers’ preferences on quantitative phenotypes. Results showed that the relative importance of wheat traits differed by gender and location. Farmer traits were variously contributed by metric traits, and could only partially be explained by them. Eventually, farmer trait values were used to produce a ranking of the 400 wheat varieties identifying the trait combinations most desired by farmers. The study scale and methods lead to a better understanding of the quantitative basis of Ethiopian smallholder farmer preference in wheat, broadening the discussion for the future of local, sustainable breeding efforts accommodating farmers’ knowledge.

## Introduction

Smallholder farms account for most part of the African farming system^1^, engaging millions of people whose subsistence depends on low-yielding agriculture exposed to increasingly erratic precipitations and climate change^2^. Modern varieties (MV) developed for high-input agriculture might not be suitable for the marginal growing conditions of smallholder farming^3–6^, as they cannot address the enormous diversity of environments and end-user needs^7–9^. In low input and high risk areas, landraces may be still chosen over MVs by local farmers^10^ because of their better adaptation, higher market value and end product quality^11,12^. Recently, genomic methods have demonstrated that traditional landraces maintained either *in situ* ^13^ or *ex situ* ^14^ contain useful allelic variation for agronomic and disease resistance traits. In these settings, the intensive cultivation of MVs with a narrow genetic base would lower the genetic diversity at a landscape scale, with detrimental effects on resilience to diseases, pests, and climate shifts^15^.

For these reasons, the top-bottom approach of centralized breeding programs in Africa struggles to produce MVs that meet local needs^16^. Participatory crop improvement methods argue instead for a bottom-up approach to better integrate farmers’ preferences into breeding^17–19^. In participatory approaches, farmers identify their preferred germplasm among collections of genotypes that are either landraces, MVs or nearly finished varieties. Farmers’ evaluations may then be joined to breeders opinions and used as an integrated selection and breeding tool^20,21^. The exploration of farmer knowledge and preferences promoted by these methods allows to understand local priorities, and by this to select or produce genetic materials with increased chances of adoption and dissemination^22,23^.

The unique diversity of Ethiopian durum wheat^14^ (*Triticum turgidum* L. subsp. *durum*) is the result of thousands of years of selection exerted by smallholder farmers^24^. Still today, more than 80% of the Ethiopian population of approximately 100 million people is engaged in agriculture^25^. Eight out of ten Ethiopian farmers are smallholders^26^, often conducting subsistence agriculture based on landraces selected for their adaptive traits to local conditions. A better understanding of the traditional knowledge guiding smallholder farmers’ choice of wheat genotypes may help in identifying and disseminating genetic materials aimed at addressing the requirements of local agriculture^27,28^.

In this study, 60 smallholder farmers from two highland communities evaluated 400 Ethiopian wheat accessions for traits of their interest. The collected information was coupled with metric measurements of 10 agronomic traits, breaking down farmers’ preferences for wheat phenotypes and identifying trait combinations contributing to farmer choice. A ranking identifying the best wheat varieties was produced upon trait values provided by farmers. We discuss the role of farmers’ traditional knowledge to identify farmer varieties to be prioritized for local breeding and distribution.

## Materials and Methods

### Experimental Sites

The study was conducted in two different locations in Ethiopia during the 2012 growing season. The first location was in the Hagreselam district, in the village of Melfa (Tigray region, 13°39′N/39°10′, 2,572 m.a.s.l.) (hereafter Hagreselam). The second location was in the Geregera area, in the village of Workaye in the Meket district (Amhara region, 11°40′N/38°52′E, 2,867 m.a.s.l.) (hereafter Geregera). The two sites are often used as test fields for the two regions of Ethiopia, and vary in altitude, temperature, rainfall, soil as well as in cultural factors, including spoken and written language (Tigrinya and Amharic, respectively). In Hagreselam, the average household size is 5.5 members on 0.6 ha. In Geregera, households are also composed by 5.5 people but occupy 1.43 ha on average. In both locations, livestock is part of the farming system, and amounts to 2.1 and 2.6 tropical livestock units (TLU) in Hagreselam and Geregera, respectively. Climatic variation at the two locations was characterized retrieving WorldClim data^29^ in the geographic information system DIVA-GIS^30^. Measures of monthly temperatures, precipitation and of 19 bioclimatic variables at Hagreselam and Geregera were analyzed with custom R^31^ scripts available upon request.

### Selection of Farmers

Thirty smallholder farmers growing wheat, 15 men and 15 women, were selected in each location and were involved in a focus group discussion (FGD) followed by a participatory evaluation (PE) in open field, held concurrently at the beginning of November. The two farmer communities are representative of the socio-economic context of Hagreselam and Geregera, in which wheat is a staple crop. Both communities already had frequent interactions with regional researchers, thus were equally experienced in dealing with participatory practices. Farmers participating in the study were chosen among volunteers residing and working in the villages where PE trials were conducted, maintaining a balance of i) gender, ii) age, and iii) wealth. Men and women alike were experienced wheat growers, and were chosen to belong to different household to avoid family bias. No specific farmer typology (*e.g*. conservative, young, leader) dominated the farmer panel. Rapporteurs to side farmer groups were chosen by the local research institutions, and they spoke the local language and were known to some of the farmers from previous interactions.

### Focus group discussions

Men and women were organized into separate groups to account for potential gender differences in wheat evaluation. At the beginning of the FGD, demographic information on the participants was collected (Supplementary Table S1). Researchers and trained facilitators who spoke the local language moderated the discussions. The FGD was aimed at: i) evaluating farmers’ perception of climate change, ii) investigating the pre-existing wheat agrobiodiversity in the two areas, iii) identifying the farmers’ selection criteria for wheat varieties.

FGD participants were asked to name the abiotic and biotic effects of climate change as well as any coping and adaptation strategies put in place. The facilitators asked eight questions listed in Supplementary Methods 1, and recorded the group’s answers.

Farmers participating to the FGD brought the wheat varieties grown in their fields, and an inventory was drawn up. Unique varieties were grouped together, and duplicates were removed. Farmers were asked to classify the genetic materials as either local varieties (landraces) or MVs, and to indicate their culinary use.

Farmers listed the traits they used to evaluate a wheat variety, which were ranked by importance. Three traits among those listed were chosen to evaluate the wheat varieties brought by the farmers and to conduct the PE: i) *earliness*, as the maturation stage of the variety at the time of the field evaluation, ii) *tillering*, as the capacity to produce secondary straws and spikes, and iii) *spike*, as the appearance and morphology of the spike. A fourth trait, *overall*, was included to represent the overall assessment of the quality of a variety. The traits were chosen because they emerged as important in both the FGDs and because it was possible to measure them in the field at the time of the PE. For each of the four traits, the participants were asked to score the wheat varieties they had brought to the FGD on a scale from one to five (1 = poor, 2 = fair, 3 = average, 4 = good, 5 = excellent) answering the following question: “What is your evaluation of [the trait] of this variety?”

### Plant materials and experimental design

Four hundred *ex situ* wheat accessions for which at least partial passport data were available were obtained from the Ethiopian Biodiversity Institute (www.ebi.gov.et). The collection is representative of the Ethiopian wheat diversity^14^: twenty-five were improved varieties, *i.e*. MVs released for cultivation. The remaining were Ethiopian wheat landraces, *i.e*. farmer varieties. Farmers involved in the PE had no previous knowledge of the varieties included in this study. To ensure uniformity, each accession obtained from the EBI was grown in an amplification field and cleaned to produce a reference variety prior to the field experiment^14^. The large number of accessions included in the PE enabled us to monitor the farmers’ preferences for genetic material they had not yet used, but which they might have access to. The wheat collection was grown under field conditions at the two locations, which are representative of different Ethiopian agroecologies. The management of the experimental fields was conducted to provide standardized growth conditions, allowing a consistent evaluation of genotypes performance across locations. Field conditions differed from those traditionally used by farmers in seed rate (higher in farmer fields) and fertilizer and weeding intensity (lower in farmer fields). Accessions were sown in four 2.5 meters rows, a seed rate of 100 kg/ha in two replications using 20 × 20 partial lattice designs. Full doses of P (100 kg/ha DAP) and half dose of N (50 kg/ha urea) fertilizers were applied at sowing. An additional half dose of N was applied at the tillering stage in both sites. Weeds were controlled manually.

### Agronomic data collection

In each location, technicians measured three phenology and seven agronomic traits. Days to 50% booting (DB), days to 50% flowering (DF), and days to 75% maturity (DM) were measured for whole plots. Number of effective tillers per plant (NET), plant height (PH, in cm), spike length (SPL, in cm), and number of seeds per spike (SPS) were measured on three randomly selected plants per plot. Grain yield (GY; grams of grain produced per plot, converted in t/ha), above ground biomass or biological yield (BY; dry weight of the above ground harvested biomass grams per plot, in t/ha) and thousand grain weight (TGW; weight of 1,000 kernels, in grams) were measured on full plots. For details and agronomic data analyses, see ref.^14^.

### Farmers’ scoring

The PE was carried out during seed maturation, when differences in phenology were still visible. In each location, the 30 farmers participating to the FGD were organized into six groups of five members each (three groups of men and three groups of women). Each group was sided by a rapporteur, a local technician with agronomic training. The rapporteur was necessary to guide the farmer group, to record farmer scores, and to prevent the emergence of dominant personalities within the group during the PE. Each group was required to independently score each PE variety for *earliness*, *tillering*, *spike*, and *overall* with the same criteria used in the FGD.

During PE, each farmer group was conducted across the experimental field, looking at each anonymously labeled plot, one after another. The groups’ points of entrance in the field were randomized. In front of each plot, the rapporteur referred to each trait under evaluation, one at a time, and asked the farmers to express their individual scores asking the question: “What is your evaluation of [the trait] of this variety?” In order to prevent farmers from influencing each other, the PE scoring was devised as follows: each farmer was given five seeds, each representing a score unit, to be held in his/her closed hand. Farmers were asked to pick a number of seeds equal to their scoring of the trait under evaluation without the other farmers seeing how many they had picked. At the signal given by the rapporteur, farmers had to open their hands all at once to show their individual scores. The scores were recorded on a datasheet following a predetermined order so that each farmer scores are traceable.

### Data analysis

An analysis of variance (ANOVA) was computed among groups for each trait using SAS/Stat® software 9.2 (SAS Institute, Cary NC). Tukey’s range test was used to verify the presence of a possible group effect and/or gender effect using SPSS® Statistics 20.0 (IBM Corp, Armonk, NY). Custom scripts in R^31^ available upon request were used to produce graphical outputs. Farmer scorings were averaged within fields according to sex groups. Men and women scores were tested for Spearman correlation in each field and for each trait separately. Parametric and non-parametric correlations were applied to scores across and within locations, and between farmers’ and rapporteurs’ scores. Spearman’s coefficients are reported. R/corrplot^32^ was used to plot the correlation analyses.

Metric measurements of phenotypes collected in each field were correlated with each other to evaluate the similarity of plant performances in the two fields. A Spearman correlation was conducted between each trait evaluated by the farmers and the most closely related metric measurement. A canonical correlation analysis^33^ (CCA) was computed with R/CCA^34^ to identify the linear combinations of metric measurements of agronomic data that best correlated with the linear combinations of the farmers’ data in each location.

PE scores were assigned weights to compile a ranking of the wheat varieties. *Overall* was given a fixed coefficient of 1, while the other traits were assigned weights following two methods. The first was based on the variability of the trait scores: the greater the standard deviation, the smaller the consistency of judgment among farmers, and the lesser the importance of the trait in the target agroecology. Traits with a higher consistency were assigned a higher weight as in Eq. (1),

1 $$W{v}_{i}=\frac{(1-c{v}_{i})}{(1-c{v}_{i})+(1-c{v}_{j})+(1-c{v}_{k})}$$

where the variation weight of a trait *i* (*Wv*_*i*_) equals the inverse to 1 of the coefficient of variation of the trait normalized to the three traits *i*, *j*, and *k*. The second method was based on the correlation among each trait *i*, *j*, *k*, and *overall*: the greater the correlation, the greater the influence of the trait over the general assessment. The correlation weights (*Wc*) were the Spearman coefficients estimated. *Wv* and *Wc* were averaged for each trait and jointly used to produce the final ranking in each PE location. Wheat varieties brought by the farmers to the FGD were also ranked accordingly. Mean phenotypic values for the two fields were calculated for each accession, and used to compute a principal component analysis (PCA) in R. The PCA was used to visualize the agronomic trait combinations in the varieties evaluated during the PE. The top 50 wheat accessions independently identified by the Hagreselam and Geregera farmers were projected onto the PCA space of the two most significant PC to evaluate the best combination of agronomic traits for the two farmer communities.

## Results and Discussion

The two gender-balanced groups of thirty farmers attended focus group discussions (FGD) and independently conducted participatory evaluations (PE) of the wheat collection in their location. On average, farmers in Hagreselam were younger that farmers in Geregera, and women were younger in both locations (Supplementary Table S1). Farmers brought to the FGD wheat varieties that they normally grew, and classified them either as *local food*, *bread* or *malt* wheat (Supplementary Table S2). Thirteen different wheat varieties were collected in Hagreselam, while 24 were collected in Geregera, demonstrating already a difference in agrobiodiversity between the two sites. Smallholder farmers generally do not make any distinction between bread and durum wheat, although they are able to differentiate the two wheat species. It is likely that most varieties listed as MV were bread wheat, whilst local food varieties were durum wheat. Farmers in the FGD were asked to prioritize traits in evaluating a wheat variety. The relative importance that farmers gave to traits depended on gender and location (Table 1). We found a general similarity in the field-related traits listed by men and women in each location. Conversely, derived traits such as suitability for drink and food preparations were perceived differently by gender. Marketability was important only for women in Hagreselam, but only for men in Geregera. The opposite occurred for local beer preparation (*tella*) (Table 1). Women in Hagreselam used a larger number of traits to evaluate wheat varieties. The difference is due to the larger number of quality and use traits adopted by women farmers (Table 1). They recognize four different uses: *enjera* (Ethiopian traditional flat bread), *bread*, *tella*, and *kolo* (toasted wheat, popular in farming and urban areas). Men in the same location are less specific and list *food preparation* as the sole food-related trait.

**Table 1:** Wheat traits identified by farmers in Hagreselam and Geregera during FGD, ordered by importance. The traits used for PE are in bold.

Rank	Hagreselam		Geregera	
	Men	Women	Men	Women
	TRAITS	TRAITS	TRAITS	TRAITS
1	Yield potential	Drought resistance	Disease resistance	**Earliness**
2	**Earliness**	Yield potential	**Earliness**	Disease resistance
3	Enjera^a^ making quality	Use	Yield potential	**Spike quality**
4	**Tillering capacity**	**Tillering capacity**	Marketability	**Tillering capacity**
5	Disease resistance	**Spike quality**	Food preparation	Frost resistance
6	Water logging tolerance	Water logging tolerance	Seed color	Drought resistance
7	Bread making quality	Height uniformity	Seed weight	Yield potential
8	Seed color	Seed size	Threshability	Water logging tolerance
9	Plant shattering	Plant height		Plant height
10	Tella^a^ making quality	Seed color		Flour amount
11		Marketability		Enjera^a^ making quality
12				Bread^a^ making quality
13				Tella^a^ making quality
14				Kolo^a^ making quality

^a^Local food/beverage.

During the FGD, farmers were required to identify climate-related threats (Supplementary Text S1; Supplementary Table S3). A shorter duration of the rainy season was one of the most important threats in both locations, and it was expectedly matched by the importance given to earliness of varieties by men and women alike (Table 1). Early varieties are more likely to escape terminal drought in the rain-fed Ethiopian wheat farming system^35^. Rainfall trends, although data series are leaky at the local level, confirm great yearly rainfall variability and spatial heterogeneity^36^, justifying the farmers’ focus on early varieties. Among the most important traits indicated by the farmers in both locations, earliness (*earliness*), tiller capacity (*tillering*) and spike morphology (*spike*) were chosen as they could be evaluated in the PE (Table 1). *Overall* was added as a measure of the general appreciation of the plot, completing the set of four PE traits.

**Figure 1:**
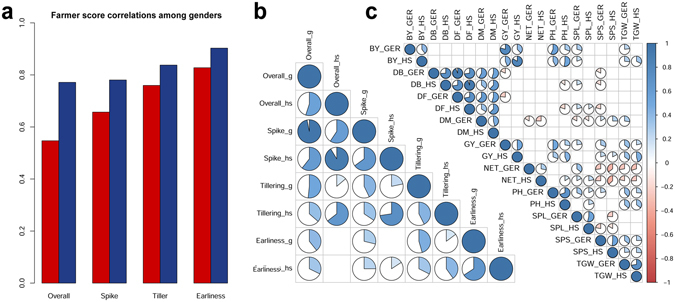
Farmer score and phenotype correlations between genders and between locations. (**a**) Score consistency between genders in the two locations. On the y axis, Spearman correlation coefficients (all significant). Red bars represent correlation values between men and women scores in Hagreselam, blue bars represent those correlations in Geregera. Gender correlations are higher in agronomic traits, *earliness* and *tillering*, and lower in quality traits, *spike* and *overall*. (**b**) PE scores consistency between the two locations (“h” for Hagreselam and “g” for Geregera). The value of Spearman’s correlations is shown by the width of pie slices colored according to the bar on the far right. The combinations without pie charts correspond to non-significant correlations. (**c**) Correlation plot of metric measures of agronomic traits in the two locations. Correlation values are shown as in panel (b), phenotype codes as in Materials and Methods. NET and BY were the agronomic traits most varied across locations.

The collection of wheat accessions used for PE was representative of Ethiopian wheat diversity, including MVs and landraces. Since the collection was maintained *ex situ* and was not previously characterized, it was devoid of researchers’ selection bias. Having the farmers analyze such a large collection of diversity increased the chances to identify landraces outperforming both MVs and materials already in farmers’ hands. At the same time, it allowed us to better understand farmers’ selection criteria. During PE, farmers provided a total of 192,000 data points. The distribution of the PE trait scores was pseudo normal except for *earliness* in Hagreselam (Supplementary Fig. S1; averages in Supplementary Table S4). This was possibly caused by the different maturing stage in which the PE was conducted in the two locations. Although phenology presented a broad variation across the 400 varieties, on average the Geregera field matured later (Supplementary Fig. S2). At the time of the PE, the mean maturing stage according to the Zadocks scale was 85 (soft dough) in Hagreselam, while in Geregera was 75 (medium milk). ANOVA revealed significant differences among farmer groups and genders (*p* < 0.001), and the distribution of PE means followed a clear gender order in Hagreselam (Supplementary Table S5). Gender-wise score correlation was lower in Hagreselam than in Geregera for all traits (Fig. 1a). The gender difference was more marked in quality and food-related traits (*overall*, *spike*) than in agronomic ones (*tillering, earliness*). Men and women participating in the experiment were selected because they were wheat growers, and were both well aware of field practices. Local differences in gender agreement were in line with the previous literature, some reporting a concordant evaluation^37^, others reporting differences^38^. There could be a very local pattern to gender disagreement, also reflected by the relative importance given to wheat traits (Table 1).

**Figure 2:**
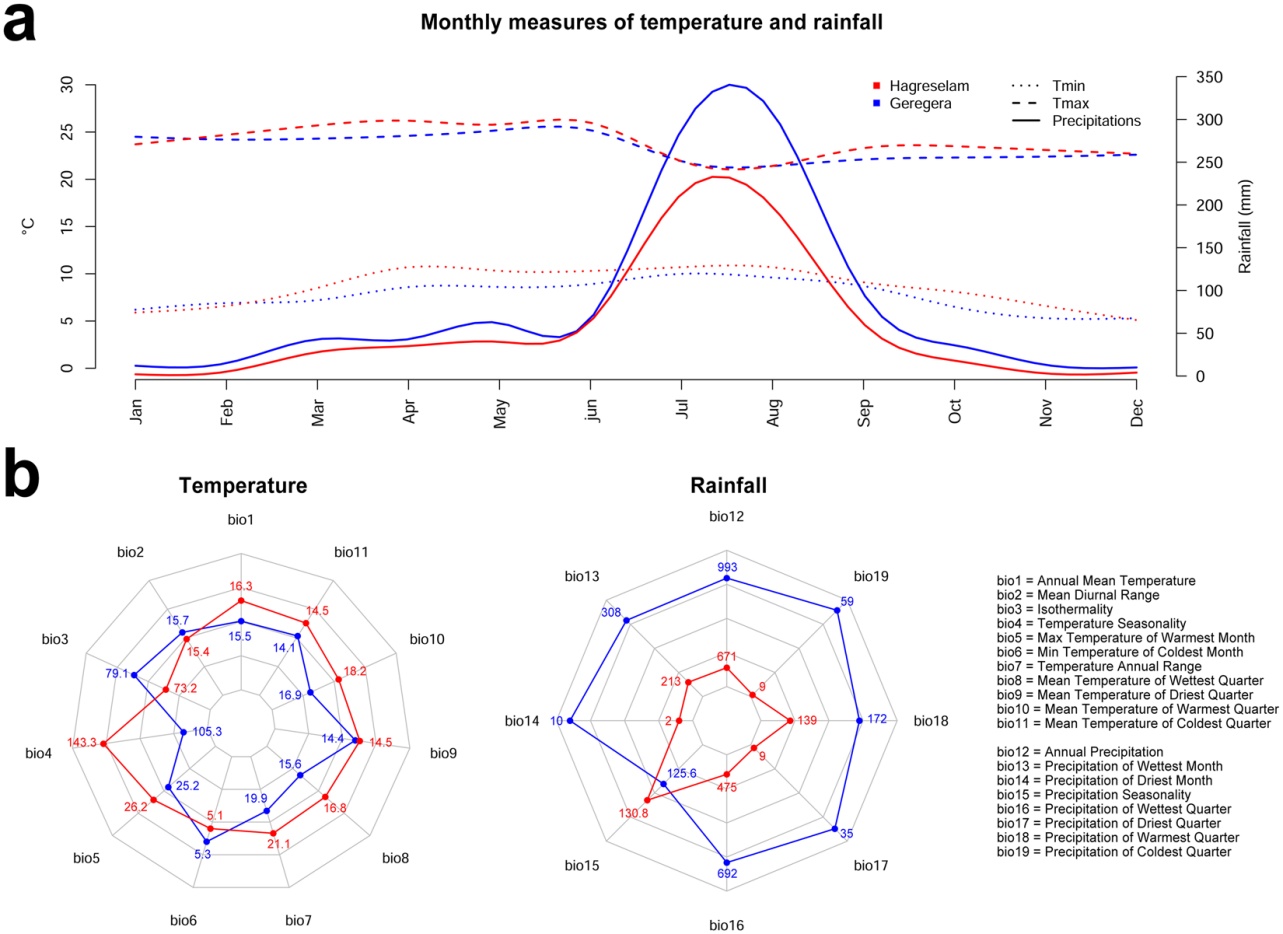
Climatic features of the experimental locations. (**a**) Yearly measures of minimum temperatures, maximum temperatures (y axis on the left side) and rainfall (y axis on the right side) in the two locations. The amount of rain and the duration of the rain season is lower in Hagreselam. Red color for Hagreselam, blue color for Geregera. Line shapes according to legend. (**b**) Bioclim values in the two locations. Among temperature indexes, seasonality separates the two locations the most. Although rainfall seasonality follows the same patterns in the two locations, Geregera is wetter across the year. Colors as in panel (a).

Although significantly correlated, the PE scores collected in the two locations held some differences (Fig. 1b). The two locations are representative of different agroecologies, and the 400 wheat genotypes showed different agronomic performances (Fig. 1b and Supplementary Fig. S2). *Earliness* and *spike* were the traits showing the greatest agreement across locations, *tillering* the lowest. NET was substantially different across locations, confirming the poor concordance of *tillering* farmer scores (Fig. 1c). Phenology was consistent across locations, with highly correlated DB, DF, and *earliness* (Fig. 1). Local agroecology may play an important role in driving farmers’ choice criteria in the two locations, which present different climatic conditions across wheat growing season (Fig. 2a). While minimum and maximum temperatures are generally stable across the year and slightly lower in Geregera, rainfall regimes diverge in the two locations. In both cases, the rain season is concentrated between June and September but rains are lower and of shorter overall duration in Hagreselam. Bioclim variables, a set of indexes deriving from interpolated monthly rainfall and temperature data^29^, help further differentiating the two PE locations (Fig. 2b). Hagreselam and Geregera differ the most in temperature seasonality and in rainfall received during the coldest and wettest part of the year. These differences may influence specific adaptation of farmer communities, and therefore affect the relative importance given to wheat traits.

To assess the relative importance of PE traits in evaluating wheat accessions, men and women PE scores were jointly analyzed. *Spike* explained most of the variability of the *overall* scores, with correlation coefficients (*r*_*s*_) above 0.9 in both locations (Supplementary Table S6). The *overall* evaluation of varieties can be considered as the farmers’ perception of the value of a specific wheat genotype, including hidden quality traits, as farmers naturally prefer material that they would grow in their own fields. Typically, the preferred varieties are those producing more grains^27^, even though other traits and trait combinations may contribute to *overall* score. Although quality traits cannot be evaluated during the PE in open field, farmers may prefer spikes and plants resembling those that in their past field experiences provided good flour types for food and drink preparations. Even though *earliness* is considered important by farmers in both locations (Table 1), this trait contributed little to none to *overall* (Supplementary Table S6). In the years prior the PE, both locations experienced an increasingly erratic rainfall (Supplementary Table S3), and this may have caused *earliness* to be considered a determinant of the harvest feasibility rather than of its quality. With such mindset, farmers may have not considered *earliness* when determining their *overall* score for any given wheat variety.

**Figure 3:**
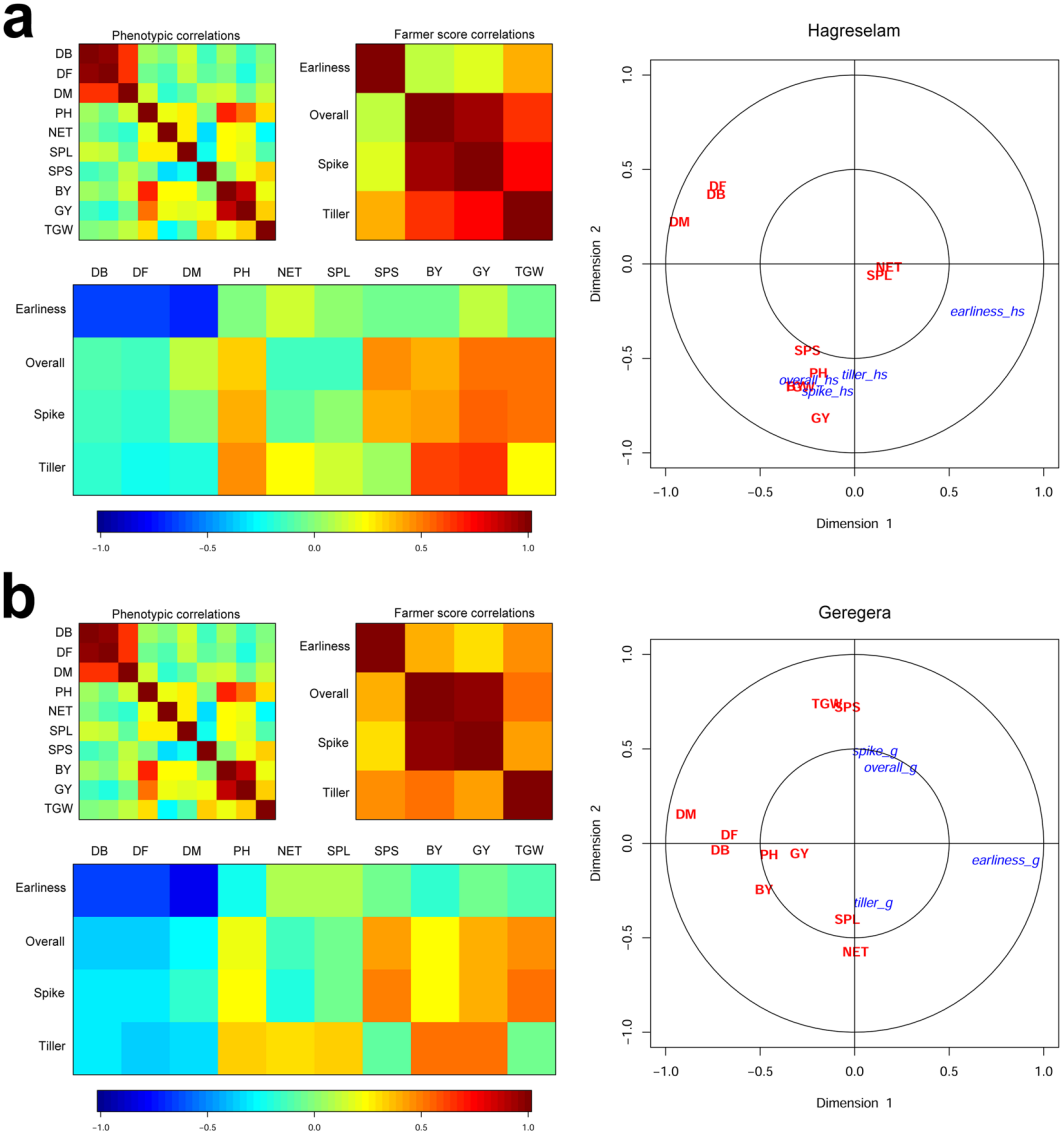
CCA reporting linear combinations of phenotypes and farmers’ evaluations in the two locations. (**a**) CCA for Hagreselam. On the left, phenotype correlations, farmer score correlations and cross-correlations according to the color scale below (blue to red, increasing correlation −1 to 1). On the right of the panel is a biplot of the original variables in the first two dimensions of the CCA space. The agronomic measures are shown in red, farmer scores are shown in blue. (**b**) CCA for Geregera, reported as in panel (a). The combination of metric traits and farmer scores is different across locations.

Linking PE with the metric measures of phenotypes is instrumental in understanding how the perception of genotype quality by farmer communities relates to the genetic material produced by breeding^39^. A canonical correspondence analysis (CCA) was used to depict the best linear combinations between PE scores and phenotypes (Fig. 3). Correlations between PE scores and analogous agronomic data are reported in Supplementary Table S7. In both locations, *r*_*s*_ between *earliness* and DB, DF and DM were highly significant, and negative. Farmers’ agreement and efficacy in measuring phenology suggests the existence of a strong selection, possibly contributed by altered rainfall patterns (Supplementary Table S3). NET showed a lower yet significant correlation with the *tillering* score (*r*_*s*_ > 0.2 in both locations), indicating a weaker preference for high tillering varieties. Tillering is a complex trait that affects the harvest index^40^. At the same time, high NET reduces sowing density and increases biomass and seedling resistance to pest attacks. The contrasting effects of NET make it liable to contrasting preferences in farmer communities (Fig. 3). This may also be due to the fact that NET shows a high variability between locations (Fig. 1c). SPS was the best indicator of spike preference (*spike*), whereas SPL was virtually not correlated with *overall* (r_s_ = −0.112). *Overall* was certainly the most composite evaluation, and was differently contributed in the two locations (Fig. 3). Metric measures of GY, BY, SPS, TGW, and PH had a positive relation with *overall* in both locations, even if higher in Hagreselam than in Geregera. PH bearing on *overall* may be explained by the fact that farmers use wheat straws for thatching their houses and feeding animals. NET and SPL, conversely, showed a poor relation with *overall* in both locations. In both locations, *spike* was highly collinear with *overall*.

**Figure 4:**
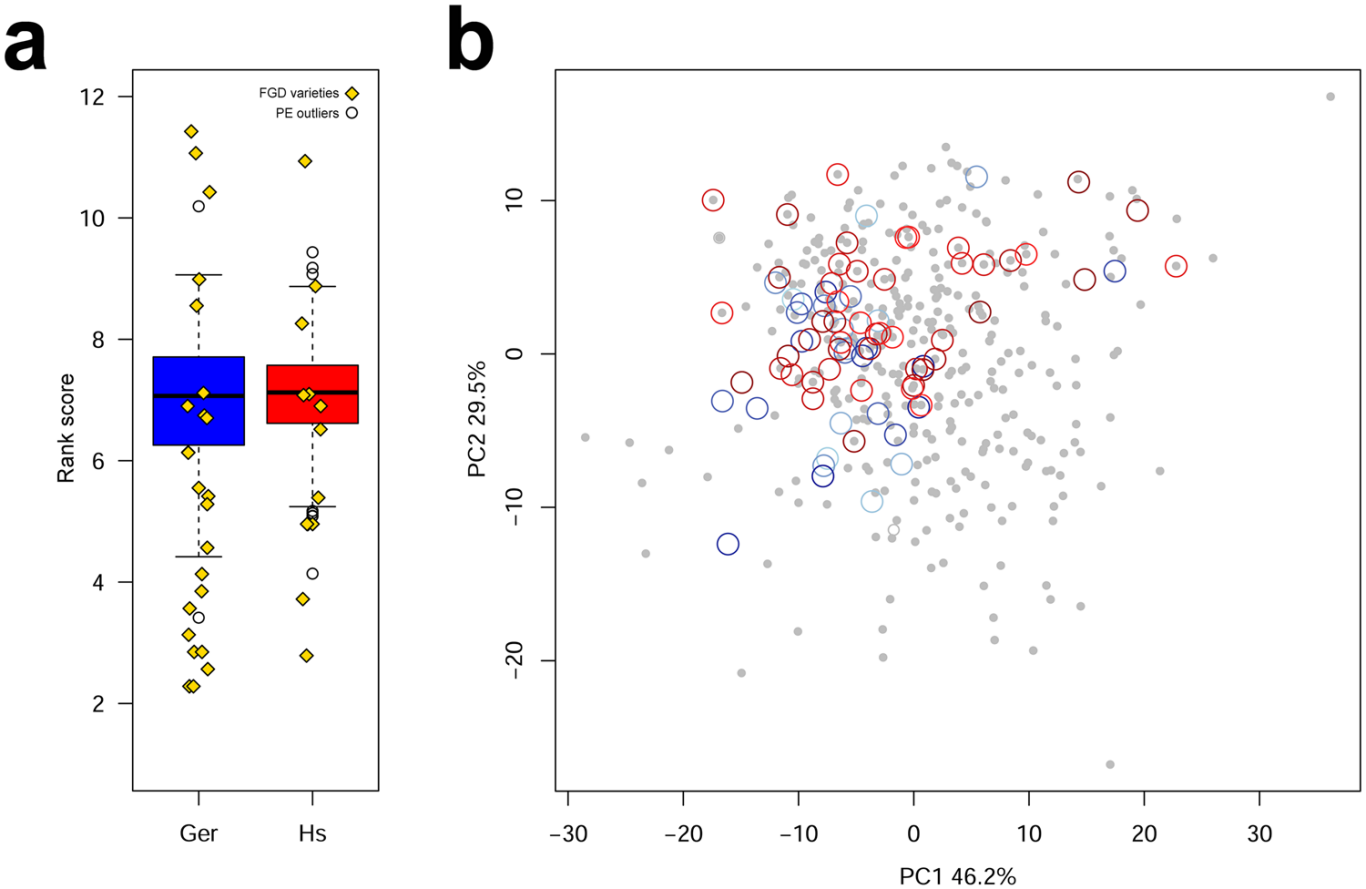
Ranking order of PE varieties. (**a**) Comparison between ranking score distributions in the two locations. Blue boxplot (Ger), Geregera; red boxplot (Hs), Hagreselam. Hollow circles outside upper and lower quartiles are outliers. Yellow diamonds are FGD varieties. (**b**) Top 50 PE wheat varieties in relation to phenotypic values. The panel depicts the first two PC axes extracted from metric measures of phenotypes averaged across the two locations. The top 50 varieties in Hagreselam and Geregera are represented by hollow circles in progressively darker red shades and blue shades, respectively. Twenty varieties were simultaneously selected in Hagreselam and in Geregera. Gray dots represent PE genotypes ranked 51 to 400. Preferred varieties in both locations are grouped in the top-left portion of the PC space.

Smallholder farmers need to be efficient in identifying the appropriate wheat genotypes to be grown in a system with very limited resilience to less-than-optimal decisions. Their traditional knowledge, which routinely guides their choice of materials, is elicited during the PE. A PE exercise on large collections of germplasm may thus be beneficial for identifying those genotypes that are worth distributing and advancing in to the breeding pipeline, engaging farmers in the breeding process. The PE scores were used to independently rank the wheat varieties in each location. Trait weights were different among locations, but retained the same order of importance: after *overall* (standard weight 1) came *spike* (0.654 in Hagreselam and 0.675 in Geregera), followed by *tillering* (0.507 and 0.479) and *earliness* (0.091 and 0.283) (Supplementary Table S8). Twenty of the top 50 varieties were common to both locations, four of which were MV: *Dinkinesh*, *Bichena*, *Tossa*, and *Workaye* (Supplementary Table S9). Although the median PE rank score was similar in the two locations, farmers in Geregera provided a wider range of scores (Fig. 4a), possibly because they were more familiar with PE practices. The same weight were also applied to rank the varieties the farmers brought to the FGD. FGD varieties ranked first in both locations, but PE varieties had a higher rank score distribution. PE evaluations given by the farmers were highly consistent: the MV *Dinkinesh* included in the PE and the MV *Dinkinesh* provided by the Geregera farmers in the FGD obtained similarly high rankings. This is especially remarkable since, while the Geregera *Dinkinesh* was evaluated during the FGD on the basis of farmers’ past experiences, the *Dinkinesh* included in the PE was blindly evaluated only by phenotypic means. The same applied to *Workaye*, the other replicated MV which ranked 29th and 3rd, respectively (Supplementary Table S9).

A PCA of the phenotypic values averaged across locations explains three quarters of the total phenotypic variance in just two PC axes, however it presents little to no structure (Fig. 4b). Each PC is a combination of several phenotypic traits (Supplementary Fig. S3), making it difficult to summarize the phenotypic diversity of the 400 accessions in a few orthogonal variables. Whereas PC1 is negatively related to yield and plant structure traits, PC2 is negatively correlated with flowering time. When the top 50 varieties according to farmer rankings were independently projected onto the PCA, it was clear that the two communities preferred the same combination of traits (Fig. 3b). Farmers from both locations co-identified 40% of the top 50 varieties (Supplementary Table S9), but even those not perfectly overlapping were in close association in the bidimensional PCA space accounting for 75.7% of the phenotypic variation. Both communities preferred wheat genotypes with slightly negative PC1 values and neutral to positive PC2 values. Farmers conducted their *overall* evaluation considering all the metric traits at once, and this combination resulted in a preferred wheat genotype that would have been difficult to derive form agronomic phenotypes individually considered.

Smallholder traditional knowledge can be gathered and related to quantitative traits, a necessary step towards the design of locally adapted ideotypes. Not only did farmers in the PE evaluate the agronomic traits that could be measured, but also and most importantly, they were driven by their own perception of the value of a wheat genotype. The originality in our approach stems from breaking down smallholder farmers’ perceptions onto metric phenotypes, eliciting traditional knowledge in agriculture with quantitative methods. Having the farmers evaluate an untapped collection of wheat diversity allowed us to survey their preference towards specific trait combinations. Farmers from the two agro-ecologies selected similar trait combinations, although they found them in different varieties. This reflects differences in wheat genotype performances in the two agroecologies, driven by different climatic conditions, and supporting the notion that local adaptation may maximize yield at any given location and favor farmers uptake of varieties^5,17^. In this phase, we preferred to increase the number of varieties tested over the number of locations to i) have a robust understanding of farmers’ selection criteria, and ii) inject novel genetic diversity into the local farming system. Surveying a broad collection of genetic diversity is also the first step towards the identification of founder lines to produce innovative breeding populations further empowering farmers’ traditional knowledge in breeding. Indeed, we anticipate that we used a nested association mapping (NAM) design^41^ to intercross 50 of the landraces evaluated in this study with an Ethiopian MV. A collection of more than 1,200 recombinant inbred lines was also evaluated by smallholder communities in Ethiopia. The information deriving from this NAM will be used to leverage advanced molecular methods in increasing the breeding value of local materials preferred by farmers. Further studies focusing on the genotype x environment interaction^42^ of the diversity panel employed here and of the genetic materials from it derived will allow the identification of accession to be prioritized in participatory breeding efforts^43^ in the considered agroecologies and beyond.

The experimental fields were conducted with a higher use of fertilizers than that typically found in farmer fields. This was necessary to allow an even evaluation of the varieties in both locations. Indeed, these fields may be considered as mother trials in which farmers identified the most desirable wheat genotypes. The top ranking varieties identified in this experiment, including several landraces topping MVs, are being redistributed and grown in fields autonomously managed by smallholder farmers with a crowd-sourcing approach^44^. By 2016, more than 900 individual households were reached in 24 villages. Focusing on a narrower set of wheat varieties on a broader collection of environments will advance our understanding of farmers’ perception of a wheat genotype value, helping to address the local needs of smallholder agriculture.

## Electronic supplementary material

supplemental figures and tables

Table S9
