# Erratum: Joining smallholder farmers’ traditional knowledge with metric traits to select better varieties of Ethiopian wheat

**DOI:** 10.1038/s41598-017-12288-5

**Published:** 2017-10-12

**Authors:** Chiara Mancini, Yosef G. Kidane, Dejene K. Mengistu, Melfa and Workaye Farmer Community, Amit, Letegabriel, Tsedal, B. Tinasu, Letekiros, Hadash, Letemedhin, A. Tinasu, Kiros, Leteberhan, Letekidan, Hareya, Letecheal, Hiritu, Ametezigu, Yohannes G. Amlak, Priest Gebre G. Slassie, Priest G. Selamma Girmay, G. Micheal Gebre, G. Slassie Mesfin, Kahsay Desta, Solomon Teklay, Haftu G. Kidan, Tesfay G. Egziabher, Priest Weldeslassie Desalegn, Hailemariam Gebre, Hiluf G. Micheal, Girmay Mebrahtu, Hailemichael, Amare Teklay, Esit Tesfay, Asrebeb Gitehun, Endale Tadesse, Mariye Asfaw, Kassaye Aragaw, Tegaye Brku, Yeshi Tadasse, Mariye Hailu, Adisse Kassun, Guzguz Gel aw, Melkam Emagn, Fenta Mitku, Asres Mengste, Bzunesh Yigzaw, Eset Tesfaw, Tesfaw Belay, Wodaje Yirga, Priest Agaju Sisay, Bewuketu Hailu, Priest Tefera Wale, Mulugeta Setegn, Tilaye Tesfie, Biset Meretie, Libay Kassie, Tegaye Biset, Yemataw Hailu, Libay Agazu, Mulatie Yigzaw, Adimasu Yigzaw, Getachew Abate, Mario Enrico Pè, Carlo Fadda, Matteo Dell’Acqua

**Affiliations:** 5 Melfa village (Kabele), Hagreselam district, Tigray, Ethiopia; 6 Workaye village (Kabele), Meket district, Geregera Amhara, Ethiopia; 1 Institute of Life Sciences, Scuola Superiore Sant’Anna, Pisa, Italy; 2 Sirinka Agricultural Research Center, Sirinka, Woldia Ethiopia; 3 Bioversity International, C/O International Livestock Research Institute (ILRI), Addis Ababa, Ethiopia; 4 Mekelle University, Department of Dryland Crop and Horticultural Sciences, Mekelle University, Mekelle, Ethiopia

*Scientific Reports* **7**:9120; doi:10.1038/s41598-017-07628-4; Article published online 22 August 2017

Due to an error during publication, the original version of this Article incorrectly listed the ‘Melfa and Workaye Farmer Community’ at the end of the author list.

This error has now been corrected in the PDF and HTML versions of the Article.

