# Supplementary material for: Joining smallholder farmers’ traditional knowledge with metric traits to select better varieties of Ethiopian wheat
Source: Sci Rep. 2017 Aug 22;7:9120. doi: 10.1038/s41598-017-07628-4 (PMC5567301; doi:10.1038/s41598-017-07628-4)
Supplement: Supplementary file 1 — supplemental figures and tables [file 41598_2017_7628_MOESM1_ESM.doc]

**SUPPLEMENTARY MATERIALS**

**TITLE**

Joining smallholder farmers' traditional knowledge with metric traits to select better varieties of Ethiopian wheat

**AUTHORS**

Chiara Mancini^1^, Yosef G. Kidane^1,2,3^, Dejene K. Mengistu^1,4^, Melfa and Workaye Farmer Community*, Mario Enrico Pè^1^, Carlo Fadda^3^, Matteo Dell’Acqua^1$^

^1^Institute of Life Sciences, Scuola Superiore Sant’Anna, Pisa Italy

^2^Sirinka Agricultural Research Center, Sirinka, Woldia, Ethiopia

^3^Bioversity International, C/O International Livestock Research Institute (ILRI), Addis Ababa, Ethiopia

^4^Mekelle University, Department of Dryland Crop and Horticultural Sciences, Mekelle University, Mekelle, Ethiopia

*A full list of the author names and affiliations within the consortium is given after the references section

^$^Corresponding Author: Matteo Dell’Acqua, m.dellacqua@santannapisa.it


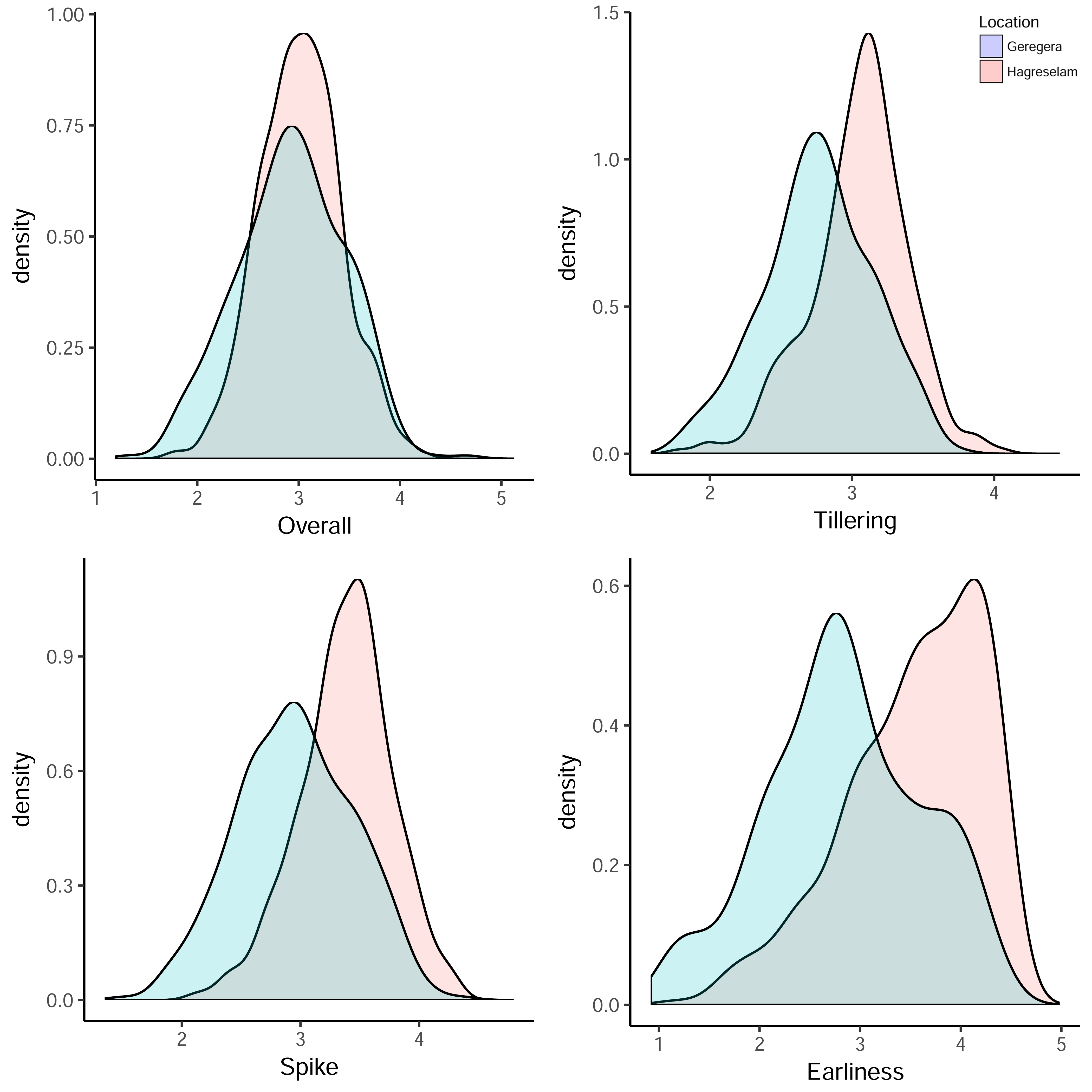
**Fig. S1.** Distribution of farmer evaluations given during PE Hagreselam (red) and Geregera (blue). The distribution is pseudo-normal for all traits except for earliness in Hagreselam, which is markedly skewed to high values. The difference in this trait evaluation across locations may be contributed by different phenology stage at the time of PE or from a different interpretation of the traits by the farmer communities.


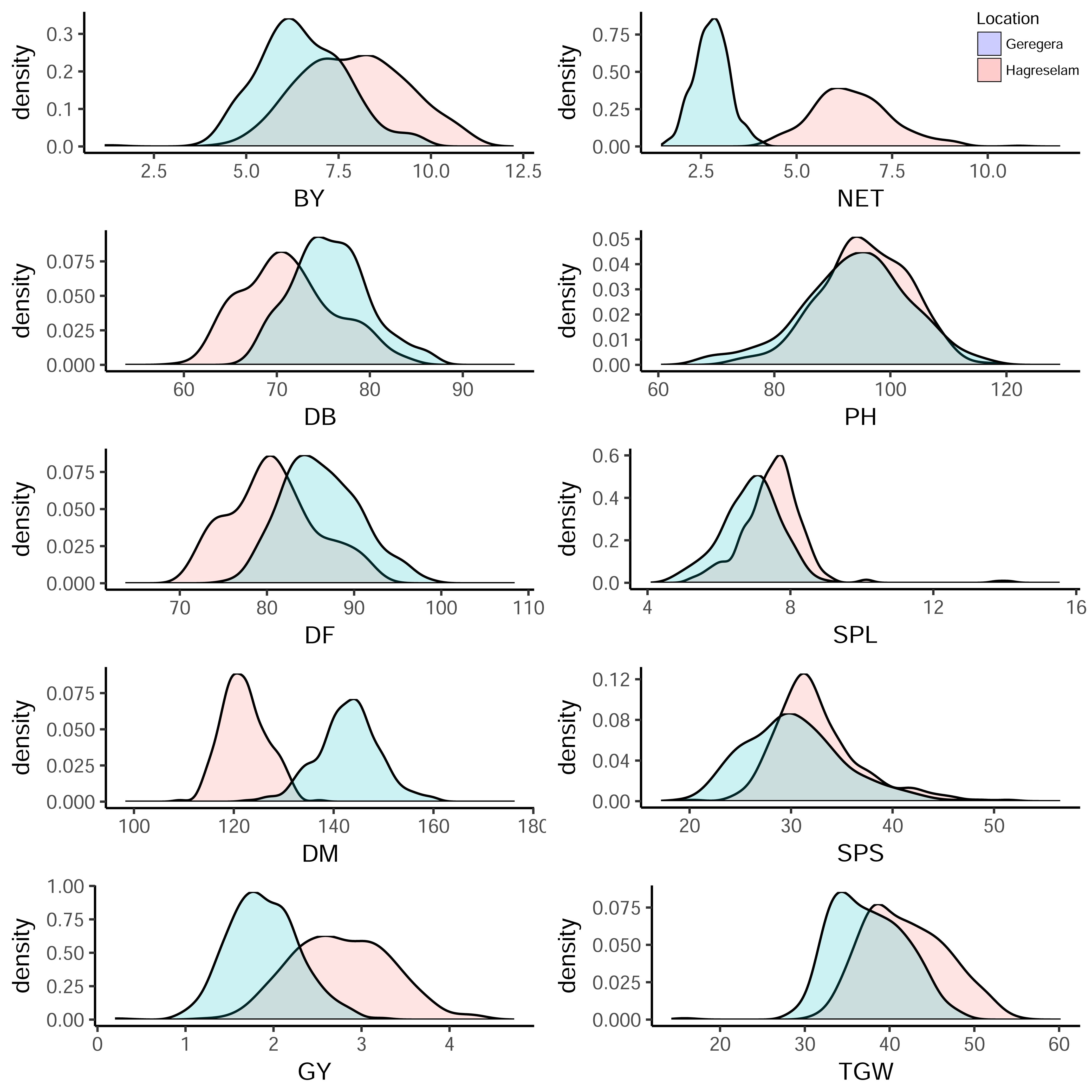


**Fig. S2.** Distribution of the metric phenotypes collected in Hagreselam (red) and Geregera (blue). The agronomic performance is similar in the two locations for biomass (BY), plant height (PH), spike length (SPL), number of seeds per spike (SPS), and thousand grain weight (TGW). Days to booting (DB), days to flowering (DF), and days to maturity (DM) are progressively delayed in Geregera. Grain yield (GY) and number of effective tillers (NET) are generally higher in Hagreselam.


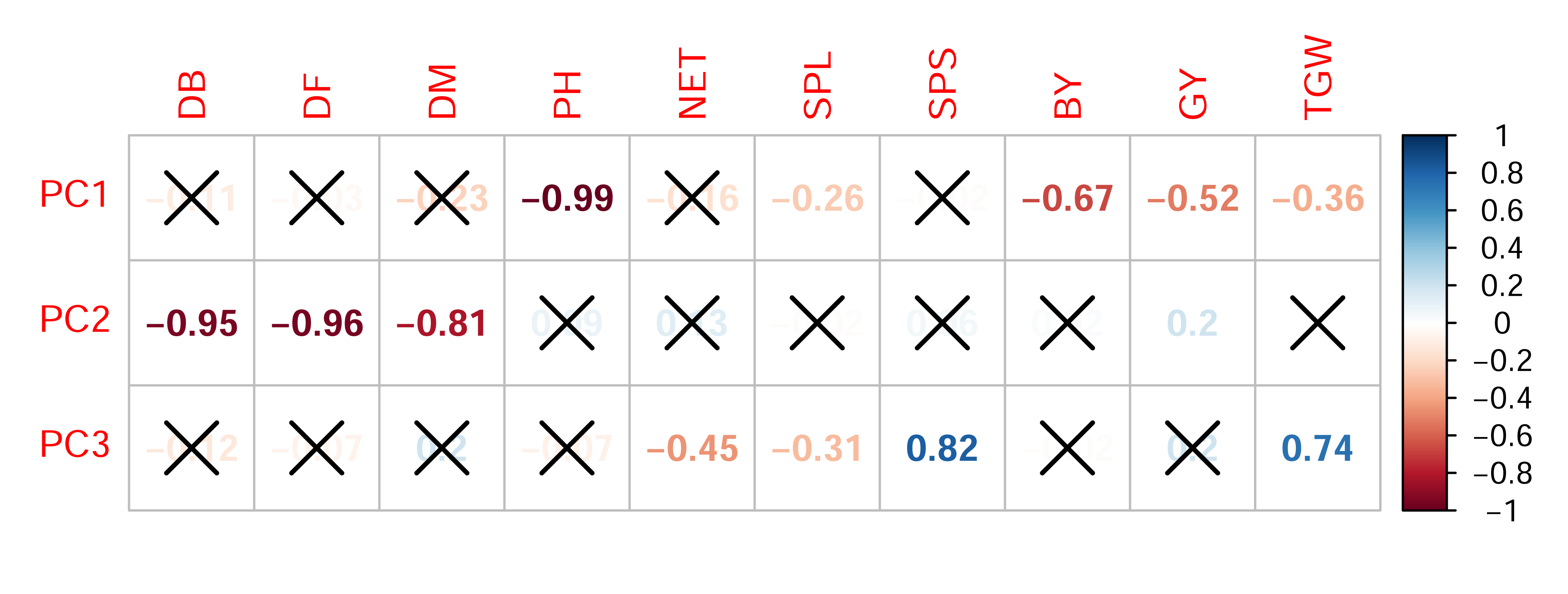


**Fig. S3.** Correlation between the original phenotypic variables and the first three axes of a principal component analysis performed on them. The value of Spearman’s correlations is shown by the numbers colored according to the bar aside. Crossed-out combinations do not provide significant correlations.

**Table S1.** Demographic information on FGD participants.

|  | Hagreselam | | Geregera | |
| --- | --- | --- | --- | --- |
|  | **Men** | **Women** | **Men** | **Women** |
| N | 15 | 15 | 15 | 15 |
| Younger | 27 | 22 | 30 | 26 |
| Older | 62 | 42 | 70 | 64 |
| Mean age | 46 | 29 | 52 | 41 |
| Std. Deviation | 10 | 6 | 12 | 10 |

**Table S2.** Wheat varieties provided by farmers in Hagreselam and Geregera during the FGD (alphabetical order). In brackets, the closest English translations of traditional names. Two MVs brought by Geregera farmers (*) were also included in the 400 accessions evaluated in the PE.

| Hagreselam | | | Geregera | | |
| --- | --- | --- | --- | --- | --- |
| **Local Name** | **Use** | **Type** | **Local Name** | **Use** | **Type** |
| *11 B* | bread | MV | *Ayfetgush* | bread-local food | local |
| *Ashmolmole* | bread | local | *Banie (bread)* | malt | local |
| *Ashmolmole melieley* | local food | local | *Borene* | bread | MV |
| *Denda’a* | bread | MV | *Debenma* | malt | local |
| *Digalo* |  | MV | *Dinknesh** | bread-local food | MV |
| *Gojo (Cottage)* | local food | local | *Furno* | bread-local food | local |
| *Gojomelieley (fill my Cottage)* | local food | local | *Gullo* | bread-local food | local |
| *Kaben* | bread | MV | *Jogole* | local food | local |
| *Melieley (fill)* | local food | local | *Kekeba* | bread-local food | local |
| *Sere’a melieley (chira feres)/Horse tail* | local food | local | *Key knkna (Red knkna)* | bread | MV |
| *Sielu* | local food | local | *Key yewusha tirs (Red Dog Teeth)* | local food | local |
| *Shehan (very white)* | bread | local | *Kubsa* | bread-local food | MV |
| *Snday awelid (girls wheat)* | local food | local | *Lastie* | bread-local food | MV |
|  |  |  | *Logawshibo (Tiriticale)* | malt | MV |
|  |  |  | *Mirt zer (improved seed)* | bread | MV |
|  |  |  | *Nech Jegolle (white Jegole)* | bread-local food | local |
|  |  |  | *Nech yewusha tirs (White Dog Teeth)* | bread-local food | local |
|  |  |  | *Tiku snde-Dawle (Black wheat- Dawule)* | local food | local |
|  |  |  | *Tikur Jegolle (Black Jegole)* | bread-local food | local |
|  |  |  | *Tikur sindie (Black wheat)* | bread-local food | local |
|  |  |  | *Tikur snde-Dega (Black wheat- Highland)* | local food | local |
|  |  |  | *Tikur snde-Shemet (Black wheat- Shemet)* | local food | local |
|  |  |  | *Workaye** | bread-local food | MV |
|  |  |  | *Wusha Tirs (Dog Teeth)* | bread | local |

**Table S3.** Climate change perception and coping strategies according to the farmers in the FGDs.

|  | **Hagreselam** | | **Geregera** | |
| --- | --- | --- | --- | --- |
| **Claim** | **Men** | **Women** | **Men** | **Women** |
| Increasing temperatures | 93% | 80% | 100% | 100% |
| Earlier onset and cessation of precipitation | 93% | 80% | 0 | 0 |
| Late onset and early cessation of rainfall | 0 | 20% | 100% | 0 |
| Increased amount of precipitation | 93% | 60% | 100% | 0 |
| Unpredictable trend of precipitation | 0 | 0 | 0 | 100% |
| Erratic distribution of precipitation | 60% | 47% | 100% | 100% |
| Adaptation strategy | irrigation, soil and water conservation | irrigation, soil and water conservation, fertilizer, improved seeds | irrigation, soil and water conservation, fertilizer, reforestation, early maturing varieties | soil and water conservation, reforestation, selling eucalyptus wood, using compost |
| Coping strategies | financial credit, change profession | change profession, migration, selling animals, reforestation, praying | using resources, helping each other, selling eucalyptus wood | migration, asking support from government, selling animals, micro credit |
| More vulnerable subjects | children, disabled, elders, poor | Children, disabled, elders, women | animals, children, elders, women | children, disabled, elders, women |

**Table S4.** Average farmer scores given to the 400 durum wheat varieties evaluated during PE, by groups (1 to 6) and locations (Hagreselam and Geregera). The farmer scores reported are the average of the five farmers in each group, either men (M) or women (W). Average of farmers scores of the same gender across groups are also given for the two locations.

|  | **Hagreselam** | | | | | | | | |
| --- | --- | --- | --- | --- | --- | --- | --- | --- | --- |
| **TRAITS** ^a^ | Group 1 (M) | Group 2 (M) | Group 3 (W) | Group 4 (W) | Group 5 (W) | Group 6 (M) | Women | Men | |
|  | n = 400 | n = 400 | n = 400 | n = 400 | n = 400 | n = 400 | n = 400 | n = 400 | |
| Earliness | 3.76 | 2.78 | 4.22 | 3.77 | 3.43 | 3.31 | 3.80 | 3.33 | |
| Spike | 3.12 | 2.86 | 4.31 | 3.49 | 3.43 | 2.93 | 3.62 | 3.13 | |
| Tillering | 2.85 | 2.62 | 3.80 | 2.95 | 3.19 | 2.80 | 3.20 | 2.89 | |
| Overall | 3.16 | 2.66 | 3.95 | 3.36 | 3.13 | 1.64 | 3.06 | 2.98 | |
|  | **Geregera** | | | | | | | |  |
| **TRAITS** ^a^ | Group 1 (M) | Group 2 (M) | Group 3 (W) | Group 4 (W) | Group 5 (M) | Group 6 (W) | Women | Men |  |
|  | n = 400 | n = 400 | n = 400 | n = 400 | n = 400 | n = 400 | n = 400 | n = 400 |  |
| Earliness | 2.10 | 3.44 | 3.04 | 2.89 | 2.74 | 2.72 | 2.90 | 2.76 |  |
| Spike | 2.62 | 3.32 | 3.00 | 3.04 | 2.80 | 2.60 | 2.92 | 2.91 |  |
| Tillering | 2.57 | 3.23 | 3.02 | 2.85 | 2.16 | 2.56 | 2.84 | 2.65 |  |
| Overall | 2.57 | 3.53 | 2.91 | 2.66 | 2.95 | 2.75 | 2.78 | 3.02 |  |

^a^ Scoring scale: 1 = poor, 2 = fair, 3 = average, 4 = good, 5 = excellent.

**Table S5**. Tukey's range test for earliness (*Earliness*), tillering capacity (*Tillering*), spike morphology (*Spike*) and general appreciation (*Overall*) scores in PE in Hagreselam and Geregera. Each farmer group (1 to 6) is composed by either men (M) or women (W)

| **Hagreselam** | | | | | | | | | | | | | | |
| --- | --- | --- | --- | --- | --- | --- | --- | --- | --- | --- | --- | --- | --- | --- |
| *Earliness* | | | | | *Spike* | | | | *Tillering* | | | *Overall* | | |
| GROUP | | MEAN | | SUBSET^a^ | GROUP | | MEAN | SUBSET^a^ | GROUP | MEAN | SUBSET^a^ | GROUP | MEAN | SUBSET^a^ |
| 2 (M) | | 2.78 | | a | 2 (M) | | 2.86 | a | 2 (M) | 2.62 | a | 6 (M) | 1.64 | a |
| 6 (M) | | 3.31 | | b | 6 (M) | | 2.93 | a | 6 (M) | 2.80 | b | 2 (M) | 2.66 | b |
| 5 (W) | | 3.43 | | b | 1 (M) | | 3.12 | b | 1 (M) | 2.85 | b | 5 (W) | 3.13 | c |
| 1 (M) | | 3.76 | | c | 5 (W) | | 3.43 | c | 4 (W) | 2.95 | c | 1 (M) | 3.16 | c |
| 4 (W) | | 3.77 | | c | 4 (W) | | 3.49 | c | 5 (W) | 3.19 | d | 4 (W) | 3.36 | d |
| 3 (W) | | 4.22 | | d | 3 (W) | | 4.31 | d | 3 (W) | 3.80 | e | 3 (W) | 3.95 | e |
|  | |  | |  |  | |  |  |  |  |  |  |  |  |
| **Geregera** | | | | | | | | | | | | | | |
| *Earliness* | | | | | | *Spike* | | | *Tillering* | | | *Overall* | | |
| GROUP | MEAN | | SUBSET^a^ | | | GROUP | MEAN | SUBSET^a^ | GROUP | MEAN | SUBSET^a^ | GROUP | MEAN | SUBSET^a^ |
| 1 (M) | 2.10 | | a | | | 6 (W) | 2.6 | a | 5 (M) | 2.16 | a | 1 (M) | 2.57 | a |
| 6 (W) | 2.72 | | b | | | 1 (M) | 2.62 | a | 6 (W) | 2.56 | b | 4 (W) | 2.66 | a – b |
| 5 (M) | 2.74 | | b | | | 5 (M) | 2.8 | b | 1 (M) | 2.57 | b | 6 (W) | 2.75 | b |
| 4 (W) | 2.89 | | b | | | 3 (W) | 3.00 | c | 4 (W) | 2.85 | c | 3 (W) | 2.91 | c |
| 3 (W) | 3.04 | | c | | | 4 (W) | 3.04 | c | 3 (W) | 3.02 | d | 5 (M) | 2.95 | c |
| 2 (M) | 3.44 | | c | | | 2 (M) | 3.32 | d | 2 (M) | 3.23 | e | 2 (M) | 3.53 | d |

^a^ Subset for alpha = 0.05.

**Table S6.** Regression and Spearman correlation coefficients between *overall* and farmer scores of *earliness*, *tillering*, and *spike*.

|  | **Hagreselam** | |
| --- | --- | --- |
|  | Regression coefficients^a^ | Spearman correlation coefficients |
| Earliness - overall | 0.000 | -0.014 |
| Tillering - overall | 0.076 | 0.594** |
| Spike - overall | 0.918 | 0.924** |
|  |  |  |
|  | **Geregera** | |
|  | Regression coefficients^b^ | Spearman correlation coefficients |
| Earliness - overall | 0.048 | 0.402** |
| Tillering - overall | 0.140 | 0.506** |
| Spike - overall | 0.940 | 0.967** |

^a^ R^2^ = 0.607.

^b^ R^2^ = 0.960.

^**^ p<0.01

**Table S7.** Correlations between farmer scores and agronomic measurements in Hagreselam and Geregera.

|  | **Hagreselam** | | | | **Geregera** | | | |
| --- | --- | --- | --- | --- | --- | --- | --- | --- |
|  |  | | | |  | | | |
|  | Earliness | Tillering | Spike | Overall | Earliness | Tillering | Spike | Overall |
|  |  |  |  |  |  |  |  |
| DM | -0.734** |  |  | 0.176** | -0.771** |  |  | -0.247** |
| NET |  | 0.268** |  | -0.054 |  | 0.261** |  | -0.030 |
| SPL |  |  | 0.029 | -0.112* |  |  | -0.050 | -0.047 |
| SPS |  |  | 0.314** | 0.331** |  |  | 0.482** | 0.442** |
| GY |  |  |  | 0.417** |  |  |  | 0.485** |
| PH |  |  |  | 0.359** |  |  |  | 0.374** |
| BY |  |  |  | 0.362** |  |  |  | 0.031 |

** p < 0.001; * p < 0.005. DM, days to maturity; NET, number of effective tillers; SPL, spike length; SPS, seeds per spike; GY, grain yield; PH, plant height; BY, biological yield.

**Table S8.** Weights attributed to farmer traits in compiling the ranking of the best varieties, by location. *Wv*, variability weights; *Wc*, correlation weights. *Overall* had weight = 1.

|  | **Hagreselam** | | |
| --- | --- | --- | --- |
|  | *Wv* | *Wc* | Final weights |
| Earliness | 0,196 | -0.014 | 0.091 |
| Tillering | 0.420 | 0.594 | 0.507 |
| Spike | 0.384 | 0.924 | 0.654 |
|  |  |  |  |
|  | **Geregera** | | |
|  | *Wv* | *Wc* | Final weights |
| Earliness | 0.164 | 0.402 | 0.283 |
| Tillering | 0.453 | 0.506 | 0.479 |
| Spike | 0.383 | 0.967 | 0.675 |

**Table S9.** Ranking of wheat varieties evaluated during PE and of wheat varieties brought to FGDs. The two locations are reported separately. For each one, the value derived from the ranking process (Rank value) is reported in descending order between the EBI identifiers/names for PE varieties, and the traditional names for FGD varieties according to their ranking. PE variety names are unique identifiers of the material available at the EBI (http://www.ebi.gov.et/).

**Supplementary Text S1.** Climate perception questionnaire administered to farmer groups during focus group discussions.

1. Have you noticed any long-term change in temperature over the last 20 years?
2. Have you noticed any long-term change in the timing of the rain over the last 20 years?
3. Have you noticed any long-term change in the pattern (amount and distribution) of rainfall over the last 20 years?
4. What do you forecast about the future state of the weather (rainfall and temperature) in your local area and generally in the country?
5. If there have been any changes, have you done anything to deal with these changes? What adaptation strategies are in place? What are your coping strategies if your adaptation strategies are not successful?
6. Who do you think is more vulnerable to climate change related hazards?
7. Are the wheat varieties you are growing performing better, worse or stable under the changing climate?
8. Do you know of other varieties that are no longer cultivated in your village/community? What is the name of those varieties? Why are they no longer grown?
